# Robotic sample changers for macromolecular X-ray crystallography and biological small-angle X-ray scattering at the National Synchrotron Light Source II. Corrigendum

**DOI:** 10.1107/S1600577521013205

**Published:** 2022-01-01

**Authors:** Edwin O. Lazo, Stephen Antonelli, Jun Aishima, Herbert J. Bernstein, Dileep Bhogadi, Martin R. Fuchs, Nicolas Guichard, Sean McSweeney, Stuart Myers, Kun Qian, Dieter Schneider, Grace Shea-McCarthy, John Skinner, Robert Sweet, Lin Yang, Jean Jakoncic

**Affiliations:** a National Synchrotron Light Source II, Brookhaven National Laboratory, Upton, NY 11973, USA; b Linac Coherent Light Source, SLAC National Accelerator Laboratory, Menlo Park, CA 94025, USA; c STMicroelectronics, Crolles, Rhône-Alpes, France

**Keywords:** NSLS-II, National Synchrotron Light Source II, AMX, FMX, LiX, automation, high-throughput, macromolecular crystallography, biological small-angle X-ray scattering

## Abstract

A correction in the paper by Lazo *et al.* [(2021). *J. Synchrotron Rad.* **28**, 1649–1661] is made.

In the paper by Lazo *et al.* (2021), there is an error in a sentence in the third paragraph. It is erroneously stated that the European Synchrotron Radiation Facility developed the FlexED8 sample-changer. However, it has been pointed out that it was the EMBL-Grenoble Instrumentation team who actually developed the FlexED8 sample-changer, as shown in the paper by Papp *et al.* (2017 ▸).

The correct sentence should read: ‘Across the pond, Diamond Light Source achieved one of the fastest sample exchange times with BART, a six-axis robotic arm system (O’Hea *et al.*, 2017 ▸), and the EMBL-Grenoble Instrumentation team developed the FlexED8, a six-axis robotic arm-based sample-changer, coupled with a novel ice-filtering dewar designed to accept several sample holders: SPINE, SPINEplus, miniSPINE and NewPin (Cipriani *et al.*, 2006 ▸; Papp, Felisaz *et al.*, 2017 ▸).’
